# The Traditional Chinese Medicine Prescription Pattern of Endometriosis Patients in Taiwan: A Population-Based Study

**DOI:** 10.1155/2012/591391

**Published:** 2012-09-26

**Authors:** Ruei-Chi Fang, Yueh-Ting Tsai, Jung-Nien Lai, Chia-Hao Yeh, Chien-Tung Wu

**Affiliations:** ^1^Institute of Traditional Medicine, School of Medicine, National Yang-Ming University, No. 155, Sec. 2, Linong Road, Taipei 112, Taiwan; ^2^Department of Chinese Medicine, Taipei City Hospital, Yangming Branch, Taipei 111, Taiwan; ^3^Department of Chinese Medicine, Taipei City Hospital, Linsen Chinese Medicine Branch, Taipei 104, Taiwan

## Abstract

*Background*. Traditional Chinese medicine (TCM), when given for symptom relief, has gained widespread popularity among women with endometriosis. The aim of this study was to analyze the utilization of TCM among women with endometriosis in Taiwan. *Methods*. The usage, frequency of service, and the Chinese herbal products prescribed for endometriosis, among endometriosis patients, were evaluated using a randomly sampled cohort of 1,000,000 beneficiaries recruited from the National Health Insurance Research Database. *Results*. Overall, 90.8% (*N* = 12, 788) of reproductive age women with endometriosis utilized TCM and 25.2% of them sought TCM with the intention of treating their endometriosis-related symptoms. Apart from the usage of either analgesics or more than one type of medical treatment, the odds of using TCM and Western medicine were similar in all types of conventional endometriosis treatment. However, endometriosis patients suffering from symptoms associated with endometriosis were more likely to seek TCM treatment than those with no symptoms. There were 21,056 TCM visits due to endometriosis and its related symptoms, of which more than 98% were treated with Chinese herbal products (CHPs). *Conclusion*. *Gui-Zhi-Fu-Ling-Wan* (Cinnamon Twig and Poria Pill) containing sedative and anti-inflammatory agents is the most commonly prescribed Chinese herbal formula mainly for the treatment of endometriosis-related symptomatic discomfort and the effects of these TCMs should be taken into account by healthcare providers.

## 1. Introduction

Endometriosis, defined as the presence of endometrial tissue at sites outside its normal location in the uterus causing pain or infertility [1–3], is among the most common reason for absence from work and school [4]. Although several studies have suggested that medical and surgical treatment are effective ways of relieving pain and preserving fertility [3, 5], endometriosis is likely to remain problematic because of the side effects associated with the chronic administration of painkillers and hormonal treatments [6–10] as well as the high recurrence rate after a surgical procedures [11, 12]. Not surprisingly, alternative therapies have become increasingly popular and are quickly approaching conventional therapy in frequency of use as a treatment for symptom relief among endometriosis patients [13, 14]. A literature review of traditional Chinese medicine (TCM) [15] found that postsurgical administration of Nei Yi pills, which was invented by a traditional Chinese doctor in China, may have comparable benefits to either gestrinone or danazol. This newly invented TCM intervention may be more effective in relieving dysmenorrhea and shrinking adnexal masses when used in conjunction with a Nei Yi enema but with fewer side effects [16, 17]. Unfortunately, information is limited regarding patterns of use of classical TCM in relation to endometriosis, which seems to be an area in which complementary and alternative medicines (CAM) have recently grown in popularity; furthermore, TCM now seems to offer an important alternative or complement to conventional health care in many Western countries [15]. In view of the above and because there is a lack of knowledge about what TCM profile is prescribed, there is a lack of direction for researchers or doctors trained in conventional medicine when, targeting endometriosis, they want to explore the potential mechanisms of TCM therapy, to assess the cost-effectiveness of using TCM therapy, and to observe the interaction between Chinese herbs and conventional therapies.

TCM, which includes acupuncture, traumatology manipulative therapies, and Chinese herbal products (CHPs), has been an important part of health care in Taiwan for hundreds of years and is fully reimbursed under the current National Health Insurance (NHI) system. Accordingly, the claims database provides a platform for understanding the utilization of TCM therapies by licensed TCM doctors [18–20]. The aim of our study is to analyze a random sample of this comprehensive database and to determine the TCM utilization patterns of women with newly diagnosed endometriosis in Taiwan. The results of this study should provide valuable information that will enable physicians to respond to the patients' use of TCM in an informed way, which in turn will further strengthen the patient-physician relationship when treating endometriosis.

## 2. Materials and Methods

### 2.1. Data Resources

This study was designed as a population-based study analyzing a sample of one million subjects selected at random from the 22 million beneficiaries of the National Health Insurance scheme of Taiwan and aimed to determine the prevalence of using prescribed Chinese herbal products among reproductive age women with endometriosis between January 1, 1998, and December 31, 2008. All data were obtained from the National Health Insurance Research Database (NHIRD), which includes all the reimbursement data of the NHI with the identification numbers of all individuals encrypted and transformed; this database is maintained by the National Health Research Institutes of Taiwan [21]. The NHIRD database contained patient's gender and date of birth, all records of clinical visits and hospitalization, prescribed drugs and dosages, including CHP, and three major diagnoses coded in the *International Classification of Diseases, Ninth Revision, Clinical Modification* (ICD-9-CM) format [22].

### 2.2. Study Subjects

**Figure 1:**
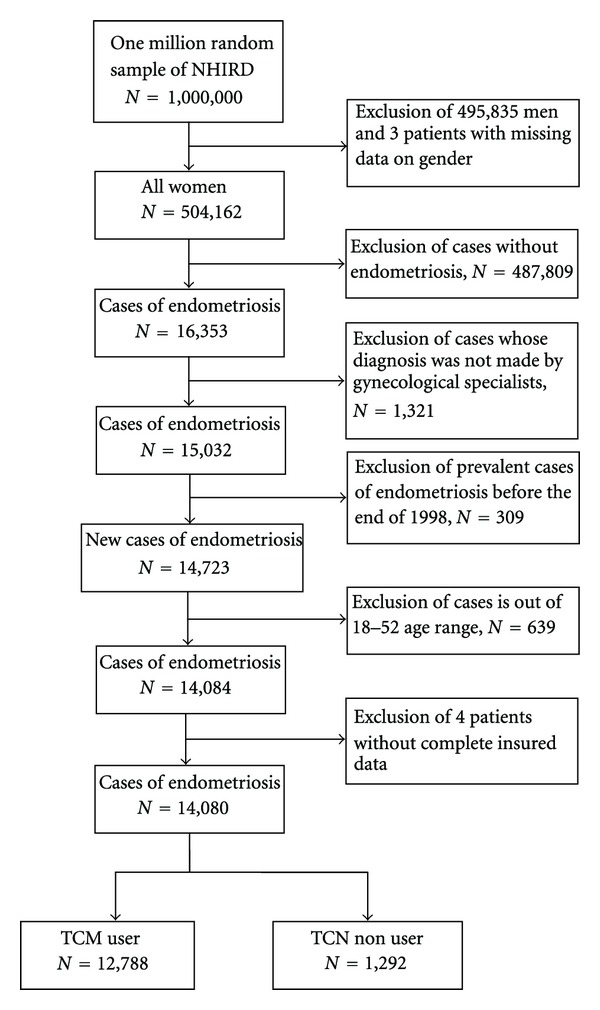
Flow recruitment chart of subjects from the one million random samples obtained from the National Health Insurance Research Database (NHIRD), 1998 to 2008, in Taiwan.

The selection of study subjects from the random sample of one million individuals was performed as follows (Figure 1). First, we excluded all male beneficiaries (*n* = 495,835) or those with missing information concerning gender (*n* = 3). Second, female beneficiaries without endometriosis (*n* = 487,809) were excluded to limit the study sample to endometriosis (ICD-9 code 617) patients. Third, the diagnosis of endometriosis that was not made by qualified gynecological specialists (*n* = 1,321), the prevalent cases of endometriosis (*n* = 309) that had been diagnosed before the end of 1998, and those without complete NHI reimbursement data (*n* = 4) were also excluded to make sure that all the subjects included were newly diagnosed with endometriosis. Fourth, subjects under 18 and over 52 years of age (*n* = 639) were excluded to limit the study sample to the women of reproductive age in Taiwan. Finally, 14,080 study subjects were included in the study cohort.

### 2.3. Study Variables

To determine the key independent variables for utilization of TCM among women with endometriosis, we selected a series of demographic factors based on previous studies [23–25]. The ages of individuals were categorized into five groups: ≤20, 21–30, 31–40, 41–50 and ≥51 years. The geographic areas of Taiwan were classified into six regions: Taipei city, Kaohsiung city, Northern region, Central region, Eastern region, and Southern region. We split the monthly wage of individuals into four levels: New Taiwan Dollars (NT$) 0, 1–19, 999, 20, 000–39, 999, and ≥40,000.

We also searched the NHIRD database for diagnosis and treatment records related to endometriosis as independent variables. The diagnosis concentrated on the clinical symptoms associated with endometriosis, including pelvic pain (ICD-9 code 789.0), dyspareunia (625.0), dysmenorrhea (625.3), and infertility (628). The endometrial implants in locations other than the uterine cavity were divided into six types: uterus (ICD-9 code 617.0), ovary (617.1), fallopian tube (617.2), pelvic peritoneum (617.3), site unspecified (617.9), and others (including rectovaginal septum and vagina (617.4), intestine (617.5), scar of skin (617.6), other specified sites (617.8)). Surgical treatments for endometriosis were divided into three types: laparoscopic surgery, surgery of the uterus, and surgery of the ovaries. The reimbursement database contained all details related to the prescription of conventional medicines for treating endometriosis-related symptoms. Then, for the final analysis, we categorised the types of preparations used as follows: analgesics, GnRH analogs, danazol, gestrinone, and progestin.

### 2.4. Statistical Analysis

Data analysis consisted of descriptive statistics, including the prescription rates of the TCM users stratified by patient's demographic characteristics, indications for the prescription of TCM, and the most frequently prescribed herbal formulas for treating endometriosis. Primary indications were classified according to their ICD-9 code. The diagnoses were coded according to the ICD-9 and grouped into a series of distinct broad disease categories. The potential effects of Chinese herbs contained in ten most commonly prescribed CHPs were grouped according to previous *in vivo* and *in vitro* studies and these are summarized in Table 4 [14]. Multiple logistic regression was conducted to evaluate the factors that correlated with TCM use. A significance level of *α* = 0.05 was selected. The statistical software SAS 9.13 was used for data management and analysis.

## 3. Results

The database of outpatient claims contained information on 14,080 women with endometriosis from 1998 to 2008. Prevalence of endometriosis on the uterus, ovary, fallopian tube, pelvic peritoneum, site unspecified, and others was 50.5%, 15.5%, 0.3%, 3.4%, 28.0%, and 2.2%. Among them, 12,788 (90.8%) endometriosis patients used TCM outpatient services at least once. Among all TCM users, 25.2% (*n* = 3,219) used TCM for the treatment of endometriosis-related symptoms. Details on the demographic distribution of TCM users and nonusers are provided in Table 1. The mean age of non-TCM users was slight higher than that of TCM users. There were more non-TCM users than TCM users who had no endometriosis-related symptoms, had an income level of NT$≧40000, or were residing in Taipei City and Northern Taiwan. However, there was no significant difference in the received surgical treatment modalities between TCM users and non-TCM users.

Adjusted odds ratios (aORs) and 95% confidence intervals (95% CIs) obtained by multiple logistic regression are shown in Table 1. Compared with the age group 21–30 years (aOR = 1.00), those aged 31 years and above were more likely to be non-TCM users. As compared with low income women (aOR = 1.00), women with a higher income level were more likely to be TCM users except those whose income level was NT$≧40000, who were more likely to be non-TCM users. After adjusting for other factors, endometriosis patients suffering from symptoms associated with endometriosis (infertility: OR = 2.73, 95% CI: 2.19–3.39; dysmenorrhea: OR = 1.52, 95% CI: 1.18–1.95; pelvic pain: OR = 2.43, 95% CI: 2.09–2.83; dyspareunia: OR = 1.20, 95% CI: 0.64–2.26) were more likely to seek TCM treatment than those with no symptoms. As compared with women without using any medical treatment (aOR = 1.00), endometriosis patients who took either analgesics (OR = 1.23, 95% CI: 1.08–1.39) or at least two types of medical treatment (2 types: OR = 1.35, 95% CI: 1.11–1.64; ≧ 3 types: OR = 2.69, 95% CI: 1.42–5.10) for relieving their endometriosis-related symptoms were more likely to be TCM users. 

Of the women visiting TCM doctors, 315,510 (79.5%) visits were treated with prescription of Chinese herbal remedies, while the rest were prescribed acupuncture and traumatology manipulative therapies. Analyses of the major disease categories for all TCM visits are summarized in Table 2, which show that “diseases of the genitourinary system” was the most common reason for using CHPs (21.4%, *n* = 67,532), followed by “symptoms, signs, and ill-defined conditions” (17.9%, *n* = 56,385), and “diseases of the respiratory system” (13.7%, *n* = 43,117).

Details of the most frequently prescribed CHPs for treating endometriosis and its related symptoms by TCM doctors are provided in Table 3, which demonstrates that *Gui-Zhi-Fu-Ling-Wan* (Cinnamon Twig and Poria Pill) was the most frequently prescribed CHP, followed by* Dang-Gui-Shao-Yao-San *(Tangkuei and Peony Powder) and *Jia-Wei-Xiao-Yao-San* (Augmented Rambling Powder). The ten most frequently prescribed CHPs are composed of Chinese herbs that are historically used to relieve endometriosis-related symptoms. The potential effects of these Chinese herbs when used to treat endometriosis and its related symptoms are summarized in Table 4 and include anti-inflammatory, antiproliferative and pain-alleviating properties.

## 4. Discussion

The prevalence of endometriosis in Taiwan over the eleven years in the study was 2.7%, which appears to be low compared with the estimates given by previous surveys [26]. Uterus and ovary were the most common sites of involvement, which is in line with previous reports [5]. We have included all patients who were newly diagnosed with endometriosis by qualified gynaecological specialists between 1998 and 2008 from a simple random sample of one million subjects among the insured general population, and the rate of insured individuals has been consistently above 96% since 1997. Therefore, we can rule out the possibility of selection bias. 

TCM as a unique traditional therapy for various ailments has been used in Taiwan for over hundreds of years, therefore, more than 90.8% of endometriosis patients ever used TCM at least once during the 11-year study period. However, the prevalence of TCM use when treating endometriosis among Taiwanese women is far below the proportion in the United States, where it has been reported that approximately two-third of endometriosis patients use CAM as part of the treatment of their endometriosis-related symptoms [25]. The difference in results between the present study and previously reported studies is probably due to disparities in the definition of endometriosis CAM treatment between patients and qualified gynecological specialists. Previous studies have collected information on endometriosis CAM treatment via self-reported questionnaires, which represent the patients' own perceptions and expectations from any healing practice that does not fall within the realm of conventional medicine. In Taiwan, TCM doctors who are involved in the treatment of endometriosis must prescribe in line with the requirements of the NHI and code accurately the diagnosis when claiming reimbursement from the NHI bureau. The present study describes only the utilization of TCM, which is therapies that are also becoming popular in many other counties. Although the present findings cannot be generalized to a comprehensive analysis of the usage of various types of CAM, the present study does use a random national-level sample that reveals the prevalence in use of TCM as prescribed by licensed TCM doctors for the treatment of endometriosis.

The present results show that the calculated prevalence of asymptomatic endometriosis was 10.8% among endometriosis population in Taiwan and that up to 40.6% of women with endometriosis never received any conventional endometriosis treatment during the 11-year followup. Women presenting with pelvic pain, dysmenorrhea, dyspareunia, and/or infertility were more likely to be TCM users than women with asymptomatic endometriosis. Women who took either analgesics or more than one type of medical treatment were also more likely to seek the TCM doctor's advice. Furthermore, regardless of the sites of involvement in endometriosis and their experience receiving different types of surgical endometriosis treatments, the choice of any of the major medical options available to women with endometriosis was not associated with the use of TCM. Possibly a fear of the side effects of any medical treatment or surgical procedure, together with the high recurrence rate of endometriosis after various conventional treatments, is a motivation for women to use TCM in Taiwan.

The present findings show that TCM users among women with endometriosis were more likely to have a higher income or to have more symptoms of endometriosis as is shown in Table 1. Diseases of the genitourinary system together with symptoms, signs, and ill-defined conditions were the two most frequent diagnoses in the disease category for TCM visits. Furthermore, endometriosis patients tended to use CHPs to deal with endometriosis-related symptoms. Acupuncture is generally used by this population mainly for diseases of the musculoskeletal system and connective tissue, which are different indications than that of women with pelvic pain. A likely explanation may be that some particular CHPs are believed by TCM users to remove blood stasis and pelvic masses and minimize toxicity, and these TCMs are usually frequently consumed by patients want to decrease the recurrence rate of endometriosis and to relieve its uncomfortable side effects, rather than use either conventional medication treatment or surgical procedures.

*Gui-Zhi-Fu-Ling-Wan* was the most frequently prescribed formula for treating endometriosis in Taiwan, as is shown in Table 3. *Gui-Zhi-Fu-Ling-Wan* has a long history of use as part of the traditional Chinese pharmacopoeia and was first documented in the classical Chinese text *Jin Gui Yao Lue* (Essential Prescriptions from the Golden Cabinet) *circa* 210 A.D. by Zhong-Jing Zhang. In the classical literature, *Gui-Zhi-Fu-Ling-Wan *is said to activate blood circulation and to remove blood stasis, which will eventually reduce the size of abdominal masses. A traditional Chinese medical diagnosis of blood stasis is reached by discerning a characteristic pattern of pelvic pain that has the quality of boring in, is fixed and is stabbing in the lower abdomen, the presence of immovable abdominal masses, and menstrual bleeding involving dark blood and dark clots, all of which are symptoms that are extremely common in patients with endometriosis. The major drawback of this syndrome-differentiation when treating endometriosis is the lack of consideration of the number, size, and location of the endometrial implants, plaques, and endometriotic adhesions in women suffering from endometriosis. Without taking into consideration the involvement in endometriosis of the surrounding organs, which by extension does not always correlate with symptom severity, a TCM doctor will prescribe the same herbal formula to women with similar symptoms. These individuals might have symptoms that are caused by different stages of endometriosis and this might lead to TCM therapies having unpredictable effects. We therefore propose that TCM doctors should assess the symptoms and stage of endometriosis more accurately. Furthermore, when assessing any response to *Gui-Zhi-Fu-Ling-Wan *treatment, they should clarify the effectiveness of the treatment at the various sites of the endometriosis. Finally, they should explain to the patient why they think the treatment should work; this is necessary prior to the design of randomized clinical trials on this type of treatment. Information from trial drugs is important, and there needs to be innovative trial designs that will help to determine the most appropriate dose and duration of treatment by CHPs using the smallest possible sample size.

Among the ten most frequently prescribed formulas for treating endometriosis, *Shao-Fu-Zhu-Yu-Tang* (Fennel Seed and Corydalis Combination), *Wen-Jing-Tang* (Tangkuei and Evodia Combination), and *Xue-Fu-Zhu-Yu-Tang* (Persica and Carthamus Combination) are said to drive out blood stasis in the lower abdomen and are very often prescribed by TCM doctors when a blood stagnation syndrome resulting from cold is diagnosed. Other commonly prescribed formulas are associated with relieving pelvic pain (*Jia-Wei-Xiao-Yao-San* or Augmented Rambling Powder; *Dang-Gui-Shao-Yao-San *or Tangkuei, and Peony Powder and *Shao-Yao-Gan-Cao-Tang* or Peony and Licorice Combination), with an irregular menstruation cycle (*Long-Dan-Xie-Gan-Tang* or Gentiana Drain the Liver Decoction and *Qui-Pi-Tang* or Ginseng and Longan Combination), or with breast swelling and tenderness (*Xiao-Yao-San* or Tangkuei and Bupleurum Formula). It is apparent from this study that TCM doctors in Taiwan prescribed herbal therapies mainly to reduce endometriosis-related symptomatic discomfort. Previous studies have revealed that some Chinese herbs have sedative and pain-alleviating properties that act via cytokine suppression and COX-2 inhibition [13, 14]. These modes of action are a reasonable explanation for the higher frequency of prescription of formulas containing Chinese herbs cytokine suppression and COX-2 inhibition in the present study. Although clinical studies on various herbs have shown promising effects and herbal medicine has been prescribed safely by professionals in the USA and Taiwan for many years, there is as yet insufficient evidence to allow a conclusion to be reached regarding the cost-effectiveness of the aforementioned formulas in relation to the apoptosis of endometriotic cells and the provision of pain relief among a population suffering from endometriosis. Further studies are warranted to assess the formula found to be generally used by TCM practitioners in this study and to determine whether they should be used as an add-on treatment for women receiving conventional treatment. It should also be assessed whether they are a potential alternative for young adults with endometriosis-associated pain syndrome who do not experience untoward side effects or have long-term morbidities caused by standard hormonal endometriosis regimens and surgical procedures.

The present study has three limitations. Firstly, this study did not include Chinese herbal remedies purchased directly from TCM herbal pharmacies, nor did we include health foods containing herbs. Thus, the frequency of CHPs utilization might have been underestimated. However, because the NHI system has a comprehensive coverage for TCM prescriptions, which is generally less costly than the cost of herbs sold in Taiwan's markets, the likelihood that subjects purchased large quantities of other herbs outside the NHI database is not high. Secondly, we are unable to draw any conclusion about the relationship between the severity of the endometriosis-related symptoms and TCM utilization for lack of actual clinical data. Lastly, our study is its retrospective nature or lack of a randomized placebo group. A previous prospective clinical trial concluded that endometriosis-treatment is associated with an important placebo effect, especially pain [27]. Thus, great caution is necessary in interpreting results of the most commonly prescribed Chinese formulas obtained in present study due to the high likelihood of a placebo effect.

## 5. Conclusions

Endometriosis patients tended to use Chinese herbal products to deal with pain-related symptoms, rather than use acupuncture. *Gui-Zhi-Fu-Ling-Wan* is the most frequently prescribed formula by TCM doctors in Taiwan for endometriosis patients. It is used with the intention of removing blood stasis and abdominal masses. Although some evidence does support the use TCM to treat endometriosis, the results from the current study could have been confounded by placebo effect, emphasizing the need for well-conducted, double-blind, randomized, placebo-controlled studies to further evaluate the efficacy of *Gui-Zhi-Fu-Ling-Wan* on women with endometriosis.

## Figures and Tables

**Table 1:** Demographic characteristics and results of multiple logistic regression showing the adjusted odds ratio (aOR) and 95% CI (confidence interval) of women with endometriosis from 1998 to 2008 in Taiwan.

Characteristics	TCM^a^ nonuser (%)	TCM user (%)	aOR^b^ (95% CI^c^)
No. of cases	1,292	12,788	
Age at diagnosis (years)			
Mean ± SD	37.6 ± 8.4	36.3 ± 8.4	
≦20	40 (3.1)	354 (2.8)	0.85 (0.59–1.22)
21–30	252 (19.5)	3,196 (25.0)	1.00
31–40	452 (35.0)	4,680 (36.6)	0.82 (0.70–0.97)
41–50	510 (39.5)	4,266 (33.3)	0.66 (0.56–0.77)
≧51	38 (2.9)	292 (2.3)	0.64 (0.45–0.92)
Insured salaries (NT$^d^/month)			
0^+^	304 (23.5)	2,394 (18.7)	1.00
1–19,999	556 (43.0)	5,770 (45.1)	1.34 (1.15–1.56)
20,000–39,999	275 (21.3)	3,436 (26.9)	1.74 (1.45–2.07)
≧40000	157 (12.2)	1,188 (9.3)	1.18 (0.95–1.46)
Insured region			
Taipei City	441 (34.1)	3,248 (25.4)	1.00
Kaohsiung City	103 (8.0)	1,110 (8.7)	1.52 (1.21–1.91)
Northern Taiwan	348 (26.9)	3,194 (25.0)	1.26 (1.09–1.47)
Central Taiwan	104 (8.1)	1,884 (14.8)	2.57 (2.05–3.21)
Southern Taiwan	270 (20.9)	3,051 (23.9)	1.61 (1.36–1.90)
Eastern Taiwan	26 (2.0)	280 (2.2)	1.55 (1.02–2.34)
Site of involvement			
Site unspecified	362 (28.0)	3,444 (26.9)	1.00
Endometriosis of uterus	653 (50.6)	6,574 (51.4)	1.09 (0.95–1.25)
Endometriosis of ovary	200 (15.5)	2,013 (15.7)	1.04 (0.87–1.25)
Endometriosis of fallopian tube	4 (0.3)	29 (0.2)	0.83 (0.29–2.39)
Endometriosis of pelvic peritoneum	44 (3.4)	415 (3.3)	1.06 (0.76–1.47)
Endometriosis of other sites	29 (2.2)	313 (2.5)	1.00 (0.67–1.50)
Endometriosis-related symptoms			
No symptoms	286 (22.1)	1,235 (9.7)	1.00
Infertility	153 (11.9)	2,100 (16.4)	2.73 (2.19–3.39)
Dysmenorrhea	96 (7.4)	671 (5.3)	1.52 (1.18–1.95)
Pelvic pain	745 (57.7)	8,714 (68.1)	2.43 (2.09–2.83)
Dyspareunia	12 (0.9)	68 (0.5)	1.20 (0.64–2.26)
Number of endometriosis-related symptoms			
0	286 (22.1)	1,235 (9.7)	1.00
1	606 (46.9)	4,989 (39.0)	1.79 (1.53–2.10)
2	347 (26.9)	5,383 (42.1)	3.28 (2.75–3.90)
≧3	53 (4.1)	1,181 (9.2)	4.56 (3.34–6.23)
Gynecological surgery for endometriosis			
No gynecological surgery	1,069 (82.7)	10,323 (80.7)	1.00
Surgeries of the uterus	84 (6.5)	783 (6.1)	1.02 (0.81–1.30)
Surgeries of the ovaries	57 (4.4)	736 (5.8)	1.18 (0.89–1.56)
Laparoscopic surgery and surgery of the uterus	23 (1.8)	267 (2.1)	1.23 (0.80–1.90)
Laparoscopic surgery and surgery of the ovaries	55 (4.3)	618 (4.8)	1.01 (0.75–1.34)
Surgery of the uterus and ovaries	3 (0.2)	37 (0.3)	1.13 (0.35–3.68)
Laparoscopic surgery and surgery of the uterus and ovaries	1 (0.1)	24 (0.2)	2.43 (0.33–18.08)
Medical treatment for endometriosis			
No medical treatment	582 (45.1)	5,133 (40.1)	1.00
Analgesics	516 (39.9)	5,910 (46.2)	1.23 (1.08–1.39)
GnRH analogs	0 (0.0)	4 (0.1)	—
Danazol/Gestrinone	72 (5.6)	636 (5.0)	0.92 (0.71–1.19)
Progestin	122 (9.4)	1,105 (8.6)	0.99 (0.81–1.23)
Number of medical treatment for endometriosis			
0	582 (45.0)	5,133 (40.1)	1.00
1	561 (43.4)	5,613 (43.9)	1.08 (0.95–1.22)
2	139 (10.8)	1,780 (13.9)	1.35 (1.11–1.64)
≧3	10 (0.8)	262 (2.1)	2.69 (1.42–5.10)

^a^TCM refers to traditional Chinese medicine; ^b^OR refers to odds ratio; ^c^CI refers to confidence interval.

^d^NT$ refers to new Taiwan dollars.

**Table 2:** Frequency distribution of traditional Chinese medicine (TCM) visits by major disease categories (according to the 9th ICD codes) in women with endometriosis from 1998 to 2008 in Taiwan.

Major disease category		Number of visits (%)		
	ICD-9-CM code range	Chinese herbal remedies	Acupuncture or traumatology	Total of TCM
Infectious and parasitic diseases	001–139	825 (0.3)	5 (0.0)	830 (0.2)
Neoplasms	140–239	3,731 (1.2)	56 (0.1)	3,787 (1.0)
Endocrine, nutritional and metabolic diseases, and immunity disorders	240–279	3,376 (1.1)	29 (0.0)	3,405 (0.9)
Mental disorders	290–319	1,629 (0.5)	14 (0.0)	1,643 (0.4)
Diseases of the nervous system and sense organs	320–389	6,431 (2.0)	525 (0.6)	6,956 (1.8)
Diseases of the circulatory system	390–459	3,807 (1.2)	43 (0.1)	3,850 (1.0)
Diseases of the respiratory system	460–519	43,117 (13.7)	205 (0.3)	43,322 (10.9)
Diseases of the digestive system	520–579	32,891 (10.4)	223 (0.3)	33,114 (8.3)
Diseases of the genitourinary system	580–629	67,532 (21.4)	499 (0.6)	68,031 (17.1)
Endometriosis	617	4,564	22	4,586
Dyspareunia	625.0	0	0	0
Dysmenorrhea	625.3	11,028	134	11,162
Infertility	628	2,634	23	2,657
Other diseases		49,306	320	49,626
Diseases of the skin and subcutaneous tissue	680–709	9,198 (2.9)	60 (0.1)	9,258 (2.3)
Diseases of the musculoskeletal system and connective tissue	710–739	13,377 (4.2)	23,160 (28.4)	36,537 (9.2)
Symptoms, signs, and ill-defined conditions	780–799	56,385 (17.9)	574 (0.7)	56,959 (14.3)
Pelvic pain	789.0	2,610	41	2,651
Injury and poisoning	800–999	1,582 (0.5)	35,597 (43.7)	37,179 (9.4)
Supplementary classification	V01–V82, E800–E999	4 (0.0)	25 (0.0)	29 (0.0)
Others*		71,625 (22.7)	20,424 (25.1)	92,049 (23.2)

Endometriosis and its related symptoms^†^		20,836	220	21,056

Total		315,510	81,439	396,949

*Other include ICD-9-CM code range 280–289, 630–677, 740–759, 760–779, and missing/error data.

^†^Endometriosis and its related symptoms include ICD-9-CM code 617, 625.0, 625.3, and 789.0.

**Table 3:** Ten most common herbal formulas prescribed by TCM doctors for the treatment of endometriosis-related symptoms among 3,219 women with endometriosis from 1998 to 2008 in Taiwan.

Herbal formula	English name	Number of person-days *N* = 319, 370 (%)	Average daily dose (g)	Average duration for prescription (days)
*Gui-Zhi-Fu-Ling-Wan*	Cinnamon Twig and Poria Pill	33,245 (10.4)	4.5	8.0
*Dang-Gui-Shao-Yao-San*	Tangkuei and Peony Powder	29,535 (9.2)	4.7	6.7
*Jia-Wei-Xiao-Yao-San*	Augmented Rambling Powder	22,876 (7.2)	4.6	7.3
*Shao-Fu-Zhu-Yu-Tang*	Fennel Seed and Corydalis Combination	21,620 (6.8)	4.7	7.0
*Wen-Jing-Tang*	Tangkuei and Evodia Combination	17,478 (5.5)	4.7	6.9
*Shao-Yao-Gan-Cao-Tang*	Peony and Licorice Combination	7,603 (2.4)	3.3	6.5
*Long-Dan-Xie-Gan-Tang*	Gentiana Combination	7,143 (2.2)	3.6	9.7
*Xiao-Yao-San*	Rambling Powder	5,502 (1.7)	4.9	7.8
*Qui-Pi-Tang*	Ginseng and Longan Combination	5,116 (1.6)	4.4	7.3
*Xue-Fu-Zhu-Yu-Tang*	Persica and Carthamus Combination	4,987 (1.6)	4.6	7.3

**Table 4:** Potential effects of the herbs contained in the ten most common herbal formulas prescribed by TCM doctors for treating endometriosis-related symptoms.

Herbal formula	Number of herbs	Ingredient herbs
*Gui-Zhi-Fu-Ling-Wan*	5	Cinnamomi Ramulus^BD^, Moutan Cortex, Paeoniae Rubra Radix^C^, Persicae Semen^D^, Poria^ABD^

*Dang-Gui-Shao-Yao-San*	6	Alismatis Rhizoma, Angelicae Sinensis Radix^ABCD^, Atractylodis Ovatae Rhizoma, Ligusticum Rhizoma, Paeoniae Alba Radix^D^, Poria^ABD^

*Jia-Wei-Xiao-Yao-San*	10	Angelicae Sinensis Radix^ABCD^, Atractylodis Ovatae Rhizoma, Bupleuri Radix^AD^, Poria^ABD^, Gardeniae Fructus, Glycyrrhizae Radix^ABD^, Menthae Herba, Moutan Cortex, Paeoniae Alba Radix^D^, Zingiberis Rhizoma

*Shao-Fu-Zhu-Yu-Tang*	10	Angelicae Sinensis Radix^ABCD^, Cinnamomi Cortex, Corydalis Tuber^BD^, Foeniculi Fructus, Ligusticum Rhizoma, Myrrha^BCD^, Paeoniae Rubra Radix^C^, Trogopterori Faeces, Typhae Pollen^D^, Zingiberis Rhizoma

*Wen-Jing-Tang*	12	Angelicae Sinensis Radix^ABCD^, Asini Gelatinum, Evodiae Fructus, Cinnamomi Ramulus^BD^, Ginseng Radix, Glycyrrhizae Radix^ABD^, Ligusticum Rhizoma, Moutan Cortex, Ophiopogonis Radix, Paeoniae Alba Radix^D^, Pinellia Tuber, Zingiberis Rhizoma

*Long-Dan-Xie-Gan-Tang*	10	Akebia Caulis, Alismatis Rhizoma, Angelicae Sinensis Radix^ABCD^, Bupleuri Radix^AD^, Gardeniae Fructus, Gentianae Radix, Glycyrrhizae Radix^ABD^, Plantaginis Semen, Rehmannia Rhizoma, Scutellariae Radix^AD^

*Shao-Yao-Gan-Cao-Tang*	2	Glycyrrhizae Radix^ABD^, Paeoniae Alba Radix^D^

*Xiao-Yao-San*	8	Angelicae Sinensis Radix^ABCD^, Atractylodis Ovatae Rhizoma, Bupleuri Radix^AD^, Poria^ABD^, Glycyrrhizae Radix^ABCD^, Menthae Herba, Paeoniae Alba Radix^D^, Zingiberis Rhizoma

*Qui-Pi-Tang*	12	Angelicae Sinensis Radix^ABCD^, Astragali Radix, Atractylodis Ovatae Rhizoma, Ginseng Radix, Poria^ABD^, Glycyrrhizae Radix^ABD^, Longan Arillus, Polygalae Radix, Vladimiria Radix, Zingiberis Rhizoma, Zizyphi Spinosi Semen, Zizyphus Fructus

*Xue-Fu-Zhu-Yu-Tang*	11	Achyranthis Radix, Angelicae Sinensis Radix^ABCD^, Aurantii Fructus, Bupleuri Radix^AD^, Carthami Flos, Glycyrrhizae Radix^ABD^, Ligusticum Rhizoma, Paeoniae Rubra Radix^C^, Persicae Semen^D^, Platycodi Radix, Rehmannia Rhizoma

^A^antiproliferative effect; ^B^sedative effect; ^C^antioxidant effect; ^D^anti-inflammatory effect.
